# Fabrication and Characterization of an Efficient Inverted Perovskite Solar Cells with POSS Passivating Hole Transport Layer

**DOI:** 10.3390/nano11040974

**Published:** 2021-04-10

**Authors:** Bo-Tau Liu, Hong-Ru Lin, Rong-Ho Lee, Nima E. Gorji, Jung-Chuan Chou

**Affiliations:** 1 Department of Chemical and Materials Engineering, National Yunlin University of Science and Technology, Yunlin 64002, Taiwan; M10615023@yuntech.edu.tw; 2 Department of Chemical Engineering, National Chung Hsing University, Taichung 40227, Taiwan; rhl@dragon.nchu.edu.tw; 3 School of Physics, Dublin City University, D09 W6Y4 Dublin, Ireland; nima.gorji@dcu.ie; 4 Department of Electronic Engineering, National Yunlin University of Science and Technology, Yunlin 64002, Taiwan; choujc@yuntech.edu.tw

**Keywords:** polyhedral oligomeric silsesquioxane, perovskite solar cell, passivation layer, NiO_x_

## Abstract

Polyhedral oligomeric silsesquioxane (POSS), featuring a hollow-cage or semi-cage structure is a new type of organic–inorganic hybrid nanoparticles. POSS combines the advantages of inorganic components and organic components with a great potential for optoelectronic applications such as in emerging perovskite solar cells. When POSS is well dispersed in the polymer matrix, it can effectively improve the thermal, mechanical, magnetic, acoustic, and surface properties of the polymer. In this study, POSS was spin-coated as an ultra-thin passivation layer over the hole transporting layer of nickel-oxide (NO_x_) in the structure of a perovskite solar cell. The POSS incorporation led to a more hydrophobic and smoother surface for further perovskite deposition, resulting in the increase in the grain size of perovskite. An appropriate POSS passivation layer could effectively reduce the recombination of the electron and hole at grain boundaries and increase the short-circuit current from 18.0 to 20.5 mA·cm^−2^. Moreover, the open-circuit voltage of the cell could slightly increase over 1 V.

## 1. Introduction

In recent years, the perovskite solar cells (PSCs) have become considered among the most promising photovoltaic materials with rapidly increasing power conversion efficiency (PCE) from 3.8 to 25.5% in a very short period of time [1], owing to lightweight, low cost, simple fabrication, high optical absorption coefficient, and large charge carrier diffusion length. Compared to conventional PSCs, inverted cells with a device structure of transparent conductive oxide/hole transport layer (HTL)/perovskite/electron transport layer (ETL)/top metal electrode feature advantages of low-temperature processability and an immense potential for developing flexible optoelectronic devices [2,3]. Within the device structure of PSCs, both HTL and ETL serve as auxiliary layers to extract the charges from the perovskite layer and to deliver them to the respective electrodes while they also block the opposite charge transfer [4,5,6] and reduce the recombination near the interfaces. Poly(3,4-ethylenedioxythiophene):poly(styrene sulfonate) (PEDOT:PSS) is commonly used for the HTL within the structure of inverted PSCs. However, PEDOT:PSS possesses insufficient electron-blocking ability, high hygroscopicity, and poor chemical stability [7,8,9]. NiO_X_, being in favor of hole transport and blocking electrons efficiently, is one of the most potential alternatives to PEDOT:PESS. Moreover, NiO_X_ leads to the higher open-circuit voltage (V_OC_) of PSCs because its work function exhibits a good alignment with valence band of CH_3_NH_3_PbI_3_ (MAPbI_3_) [10]. To reduce interfacial loss, a passivation layer has been inserted between the perovskite layer and the charge transport layer to decrease the interfacial defects and charge recombination [11,12,13]. Recently, several methods and materials, such as ultraviolet ozone [14], sodium dodecylbenzenesulfonate [15], and polystyrene [16], have been reported to passivate NO_X_ and improve the crystal size of perovskite layer. Although they revealed enhanced efficiencies, few studies were reported about how to improve the NO_X_ HTL for the fabrication of efficient PSCs. It remains a challenge to develop new passivation methods for the PSCs with NO_X_ imported as HTL in their structure.

Polyhedral oligomeric silsesquioxane (POSS) possesses a hybrid structure of inorganic siloxane cage associated with organic groups. Combining hybrid and hollow characteristics, POSS exhibits special mechanical, electrical and optical properties as important building blocks for biomedical and optoelectronic materials [17,18]. The electron-withdrawing POSS cage potentially facilitates the dissociation of Li ions to increase the electrical conductivity of nanocomposite electrolytes [19]. POSS can block the undesirable anion exchange reactions to improve the water resistivity and surface coverage of CsPbX_3_ and be used as a hole blocking layer to balance the electron–hole injection of light-emitting devices [20,21]. Liu et al. used POSS with amino group to passivate perovskite layers through coordination and hydrogen bonding between amino groups and Pb ions. The passivation increased V_OC_ and PCE owing to the decrease in the charge trap density and trap-state energy level [22]. The hydrophobic nature of POSS also improved the humidity tolerance of perovskite materials [23]. In this study, POSS was used in attempt to passivate the NO_X_ HTL in a PSC structure through its hydrophobic nature and ability of trap suppression. Here, the PSCs have been fabricated with the inverted structure of fluorine-doped tin oxide (FTO)/NO_X_/POSS/MAPbI_3_/PC_61_BM/Bathocuproine (BCP)/Ag structure. The effect of POSS content on photovoltaic properties has been analyzed using different microscopy and spectroscopy techniques. Enhanced surface hydrophobicity and flatness were induced by the POSS passivation to significantly increase the grain size of perovskite. For an optimized concentration of 0.01-mg·mL^−1^ POSS, the short circuit current (J_SC_) increased from 18.0 to 20.5 mA·cm^−2^, an increase of 13.9%. This enhancement was mainly due to the decrease in grain boundaries and the resistance of the charge recombination.

## 2. Materials and Methods 

### 2.1. Materials

FTO-coated glass substrates (7 Ω·sq^−1^) were purchased from Ruilong optoelectronics, Taiwan. Nickel(II) initrate hexahydrate (99.9985%), ethylene diamine (EDA, 99%), and ethylene glycol monomethyl ether (2-ME, 99%) were purchased from Alfa Aesar (Ward Hill, MA, USA). Acryloisobutyl POSS was received from Hybrid Plastics Inc. (Hatteriesburg, MS, USA). Methylammonium idodide (MAI), lead(II) iodide (PbI_2_), and bathocuproine (BCP, 99.5%) were purchased from Xi’an Polymer Light Technology (Xi’an, China). 6,6-Phenyl-C61-butyric acid methyl ester (PC_61_BM) were purchased from Solenne BV (Groningen, Netherlands). Anisole was purchased from Acros Organics (Geel, Belgium). Anhydrous solvents such as dimethyl sulfoxide (DMSO), dimethyl formamide (DMF), chlorobenzene (CB), and isopropanol (IPA) were purchased from Sigma Aldrich (Saint Louis, MO, USA) and used without further purification.

### 2.2. Synthesis of NO_X_ Solution

The amount of 0.87 g nickel nitrate hexahydrate and 0.12 g of EDA were mixed into 5 mL of 2-ME. The solution was sealed with parafilm and stirred overnight at 550 rpm and 60 °C. The color of the solution gradually changed from dark green to dark blue, resulting in a 0.6 M NO_X_ solution.

### 2.3. Device Fabrication

**Figure 1:**
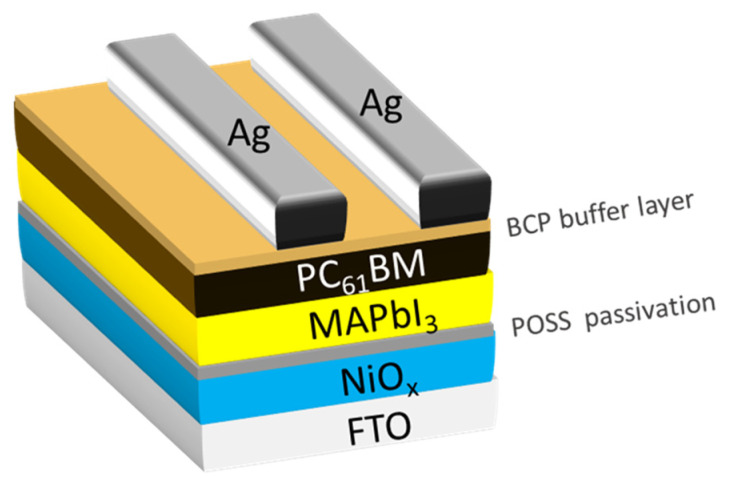
The schematic representation of structure of designed perovskite solar cells (PSCs).

The fluorine doped tin oxide (FTO) glass substrates (2 × 1.5 cm^2^) were masked by polyimide tape as T-shape pattern. Then, the unmasked portion of the FTO was etched with zinc powder/HCl solution (6 M) and then cleaned with a detergent to remove the surface residue and zinc powder, following ultrasonically with ethanol, isopropanol, and deionized water for 15 min, respectively. The fabrication process of PSCs was illustrated in Figure S1. Typically, the NO_X_ solution was spin-coated on the cleaned FTO glass substrates at 4000 rpm for 40 s and then heated at 100 °C for 10 min, followed by 300 °C for 1 h. 80 μL of POSS IPA solution in the concentration of 0, 0.005, 0.01, 0.015, and 0.05 mg·mL^−1^, was spin-coated on the as-prepared NiO_X_ substrates at 6000 rpm for 20 s and then heated at 100 °C for 10 min, respectively. MAPbI_3_ was prepared by dissolving 0.2305-g MAI and 0.6684-g PbI_2_ in DMSO/DMF in the volumetric ratio of 1:4 and then stirring the solution for one day. Sixty microliters (60 μL) of the MAPbI_3_ solution was spin-coated on the POSS-passivated substrates at 4000 rpm for 25 s in a glove box. Nine seconds (9 s) after the start of the spinning, 500 μL of anisole was dropped upon the MAPbI_3_-coated substrates as an anti-solvent. The substrates were then heated at 100 °C for 12 min to complete the film formation. Ten microliters (10 μL) of PC_61_BM CB solution was spin-coated onto the perovskite layer at 3000 rpm for 20 s. Then, the samples were heated at 80 °C for 10 min. Eighty microliters (80 μL) of BCP IPA solution was spin-coated upon the PC_61_BM layer at 5000 rpm for 20 s. Finally, a silver layer with 100 nm thickness was formed on the surface of the devices through thermal deposition under a vacuum of 5 × 10^−6^ torr and a plating rate of 0.8–0.9 Å/s. The structure of the designed devices in this study is shown in Figure 1. 

### 2.4. Measurements and Characterization

The crystalline phase of the perovskite crystals was characterized through X-ray diffraction (XRD) using an X-ray diffractometer (Miniflex II, Rigaku, Tokyo, Japan) and CuKα radiation (wavelength 0.15418 nm) with a fixed operating voltage of 30 kV and a fixed current of 15 mA. The morphology of the perovskite layers and devices were examined using a field-emission scanning electron microscope (AFE-SEM, Zeiss Auriga, Oberkochen, Germany). The absorption and photoluminescence (PL) emission spectra of the perovskite crystals were determined using a UV–Vis spectrometer (V770, Jasco, Tokyo, Japan) and a fluorescence spectrometer (LS-55, Perkin Elmer, Waltham, MA, USA), respectively. The contact angle of the POSS-passivated surface was determined using a contact angle analyzer (Phoenix 10, SEO, Suwon, Korea). The photocurrent density–voltage (J–V) characteristics were measured under irradiation of 100 mW·cm^−2^ using a solar simulator (MFS-PV, Hong-Ming Technology, Taiwan) equipped with a source meter (Keithley 2400, Keithley Instruments, Cleveland, OH, USA). Electrochemical impedance spectra (EIS) were measured over the frequency range of 50 mHz to 1 MHz with a potential perturbation of 10 mV using an electrochemical workstation (Zennium, Zahner, Kansas City, MO, USA). The incident photon-to-electron conversion efficiency (IPCE) spectra of cells were measured using an external quantum efficiency measurement system (QE-R, Enli Technology, Taiwan).

## 3. Results and Discussion

**Figure 2:**
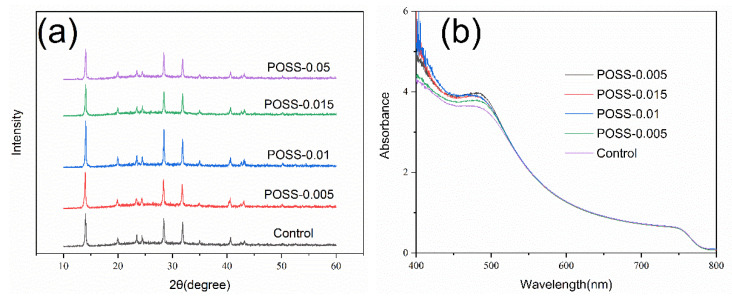
(**a**) XRD patterns and (**b**) UV–Vis absorption spectra of the MAPbI3 layers over the fluorine-doped tin oxide (FTO)/NiO_X_/POSS with various POSS contents.

The XRD patterns of the perovskite layers with various POSS contents have been measured under dark standard conditions at room temperature and presented in Figure 2a. All samples exhibited three sharp reflection peaks at 14.1°, 28.4°, and 31.8° corresponding to crystal planes (110), (220), and (310) of tetragonal I4/mcm MAPbI_3_ perovskite structure, respectively [24]. These XRD results are in agreement with XRD patterns reported in the literature [25,26]. No peak has been recorded near 12.7° which is usually attributed to PbI_2_ formed in the perovskite layer [27]. This result implies that POSS incorporation neither hinders the formation of MAPbI_3_ crystal nor results in PbI_2_ separated out from MAPbI_3_ layer. In particular, the XRD peak related to plane (110) recorded for the sample POSS-0.01 shows the highest intensity compared to other samples, indicating that 0.01-mg·mL^−1^ POSS incorporation leads to the best crystallinity. Figure 2b shows the UV–Vis absorbance of the FTO/NiO_X_/POSS/MAPbI_3_ samples with various POSS incorporation. All of curves display the same cut-off edge at 779 nm but different absorption intensity in the range of 400~530 nm. Introduction of POSS over the NO_X_ layer improves the photo-absorption of MAPbI_3_ and the strongest absorption rate is related to sample POSS-0.01.

**Figure 3:**
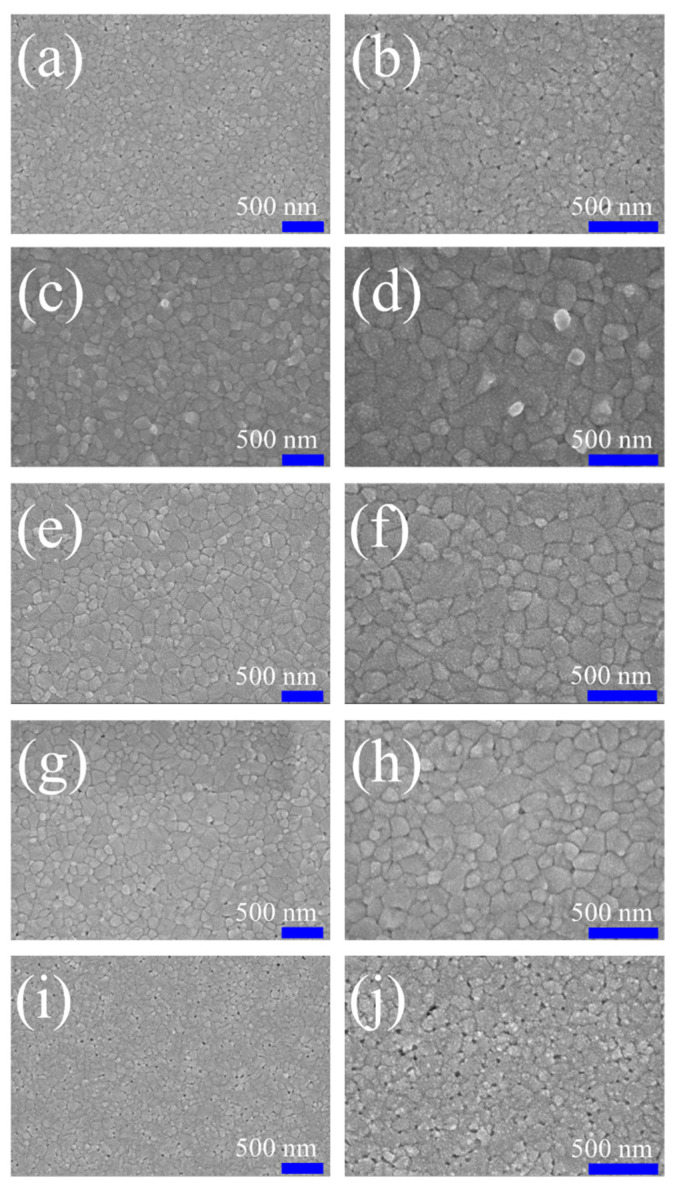
SEM images of the MAPbI_3_ layers over the FTO/NiO_X_/POSS with various POSS contents: (**a**,**b**) control; (**c**,**d**) POSS-0.005; (**e**,**f**) POSS-0.01; (**g**,**h**) POSS-0.015; and (**i**,**j**) POSS-0.05. The magnification in the left images and the right images are x30k and x50k, respectively.

**Figure 4:**
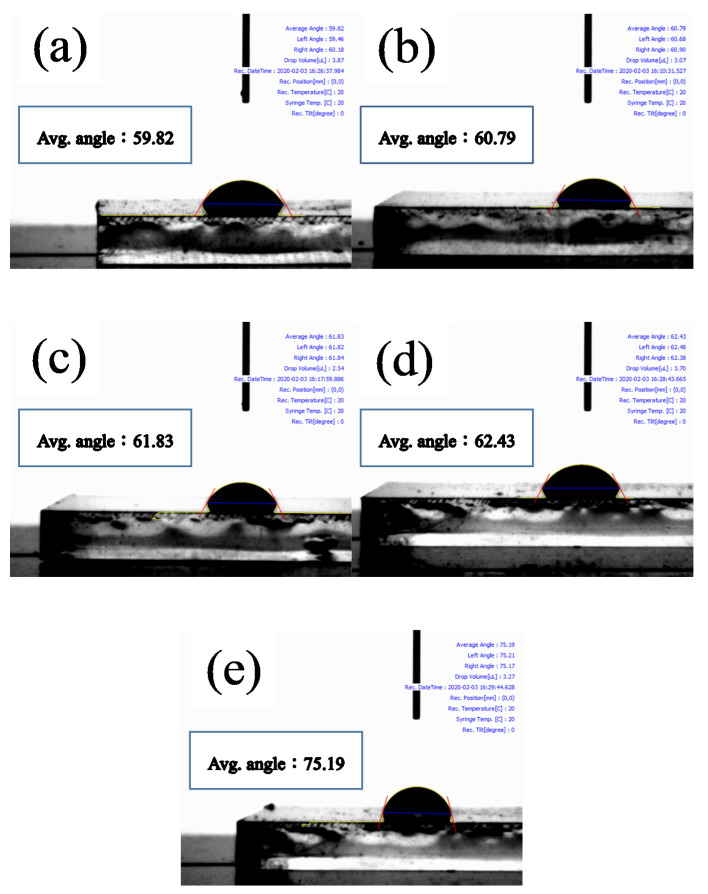
Drop images of the MAPbI_3_ layers over the FTO/NiO_X_/POSS with various POSS contents and their corresponding contact angle: (**a**) control; (**b**) POSS-0.005; (**c**) POSS-0.01; (**d**) POSS-0.015; and (**e**) POSS-0.05.

**Figure 5:**
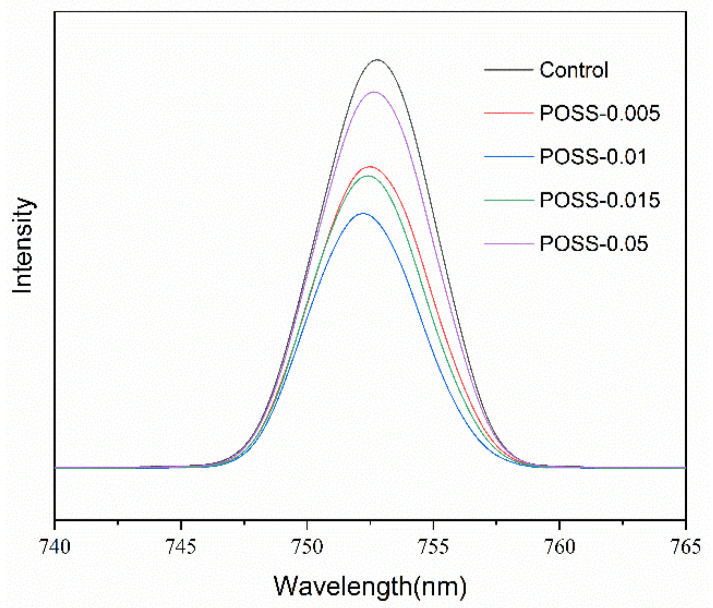
Photoluminescence spectra (PL) spectra of the FTO/NiO_X_/POSS/MAPbI_3_ with various POSS contents.

Figure 3 presents the SEM surface morphology images of the MAPbI_3_ layers deposited over POSS layers fabricated with various POSS contents. The images were recorded at two kinds of magnification. Some pinholes can be observed on the surface of the pristine perovskite layer. When the ultra-thin POSS passivation layer is introduced, the number of pinholes is reduced and reaches the minimum level at concentration of 0.01-mg·mL^−1^ POSS, which means that the coverage of the perovskite layer is more complete at this optimum POSS concentration. When the POSS concentration increases over 0.01 mg·mL^−1^, the number of voids increases again. In addition to the pinholes, the POSS concentration also influences the grain size of MAPbI_3_. When the concentration of POSS increased from 0 to 0.01 mg·mL^−1^, the crystal growth of MAPbI_3_ progressively accelerated and the grain size of perovskite enlarged from 162.3 to 226.9 nm. However, grain size decreased to 167.5 nm when the POSS concentration increased to 0.05 mg·mL^−1^ (Figure S2). We inferred that too high and too low concentration of POSS may have a negative impact on the morphology and crystallinity of the perovskite layer. Figure 4 reveals the contact angle of water drops on the surface of FTO/NiO_X_/POSS with various POSS contents. It can be realized easily that the contact angle increases with POSS amount due to the hydrophobic nature of POSS. The hydrophobic property may reduce the damage of adsorbed water and hydroxyl groups on the surface of ETL to MAPbI_3_ crystal growth. Too many POSS may make the perovskite solution difficult to spread over the surface of substrates, resulting in pinholes and small grain size. The SEM analysis is in good correlation with the XRD and UV–Vis observations where all of them recommend that an optimum amount of POSS is a key to induce optimum crystal growth. A compact perovskite film with larger grains can be obtained at optimum POSS concentration with a full surface coverage (Figure 3e,f). Comparing with the pristine NiO_X_ layer, an appropriate POSS deposition decreases the surface roughness (Figure S3). As a result, the increase in grain size of perovskite may be due to the fact that the POSS passivation makes the surface of the NiO_X_ layer more hydrophobic and smoother. The photoluminescence spectra (PL) of the MAPbI_3_ grown over the substrate with various POSS amounts present a dominant peak around 753 nm under 500 nm excitation (Figure 5). Perovskites have a direct bandgap, thus this peak can be assigned to radiative recombination. The POSS-0.01 shows a slight blue shift to smaller wavelengths around 752 nm and also the PL intensity decreases. This represents the rapid transfer of the excited electrons into ETL which is due to improved grain size and improved film morphology with reduced pinholes.

**Figure 6:**
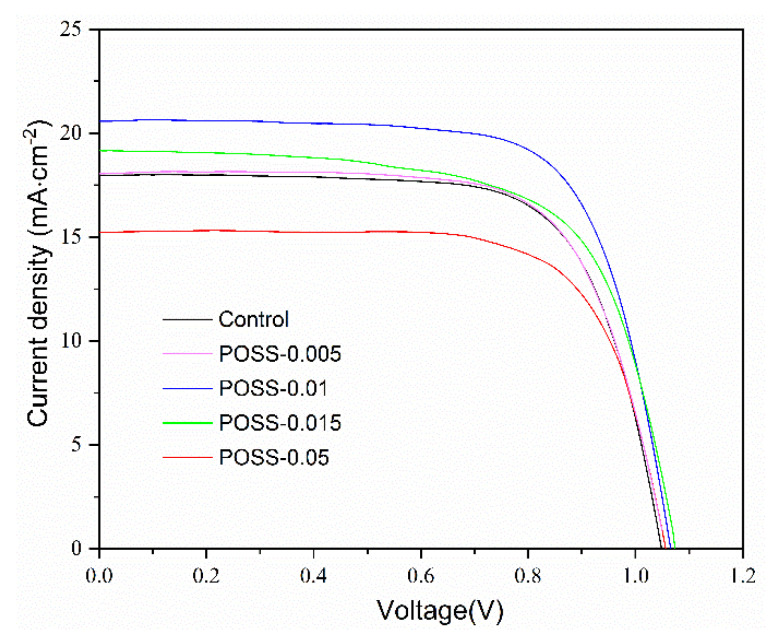
Current–voltage characteristics of the control cell and the PSC fabricated with various POSS contents.

**Figure 7:**
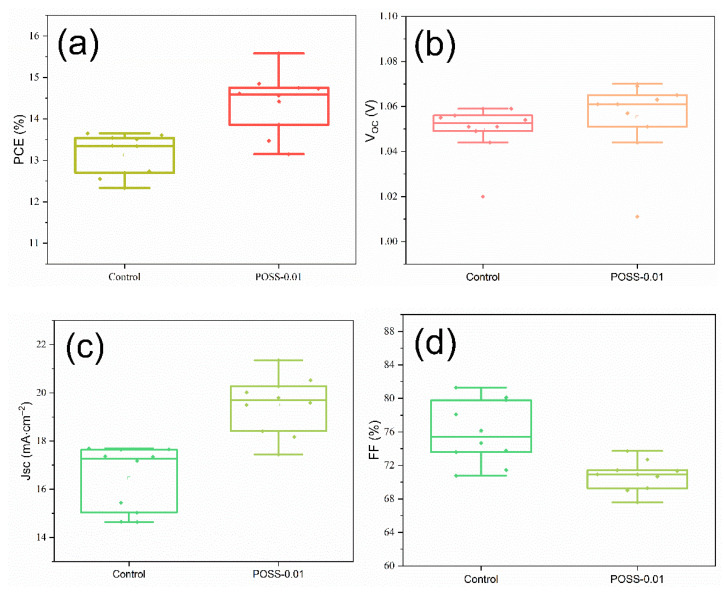
Distribution of device metrics for 10 cells fabricated without and with POSS passivation layer: (**a**) PCE; (**b**) V_OC_; (**c**) J_SC_; and (**d**) FF.

Figure 6 displays the current–voltage characteristics of the PSCs with various POSS contents for passivation. The corresponding characteristic properties are summarized in Table 1. The PCEs and current densities of the PSCs increase with increasing POSS content, reaching a maximum at POSS-0.01, and decrease with further increasing POSS content (POSS-0.05). The order is reciprocal to that of the PL intensity but follows that of grain size. As the decrease in the PL intensity of MAPbI_3_ indicates the reduction in the rate of electron–hole recombination, the enhancement on PCEs and current density caused by the POSS passivation may result from the large grain size and few pinholes to reduce the charge recombination. Compared with pristine PSC (control), the POSS-0.01 increases the PCEs and the photocurrent density from 13.3 to 15.6% and from 18.0 to 20.5 mA·cm^−2^, an increase of 17.3 and 13.9%, respectively. Figure 7 presents the variations of device parameters (V_OC_, J_SC_, fill factor (FF), and PCE) of the control cell and the champion cell POSS-0.01. The individual data, average value, and standard deviation are shown in the figures. The higher average PCE and Jsc were obtained for POSS passivated cells. However, the average FF is lower for the POSS passivated cell which is probably due to increased series resistance of the passivated cells. However, the V_OC_ of the POSS-0.01 is slightly higher than that of the control cell, responding the fact that lowering the charge recombination rate would lead to increasing V_OC_ [28,29].

**Figure 8:**
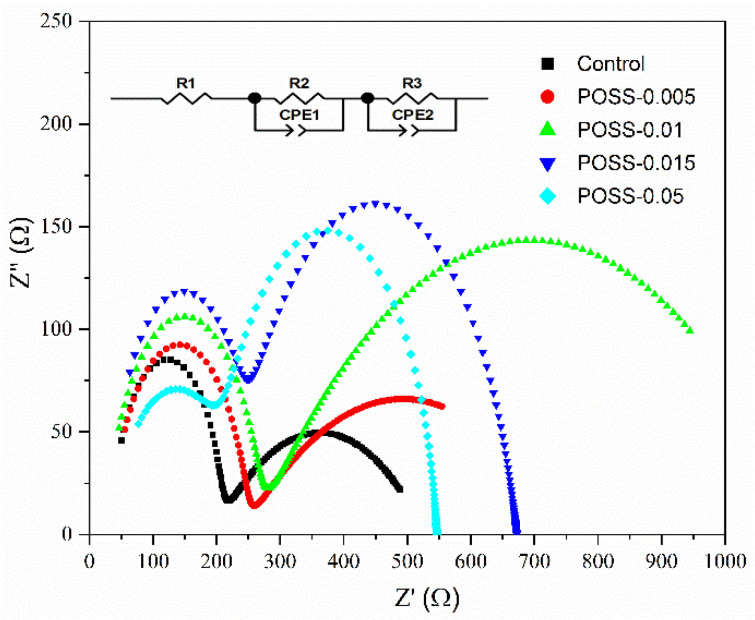
Nyquist plots of the electrochemical impedance spectra (EIS) of the control cell and the PSC fabricated with various POSS contents.

**Figure 9:**
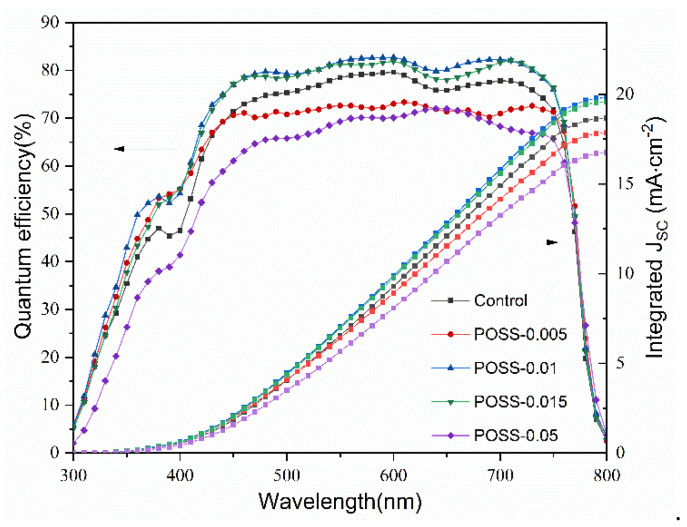
IPCE spectra and integrated J_SC_ of the control cell and the PSCs with various POSS contents.

The Nyquist plots (Z′–Z′′) of the EIS of the PSCs with various POSS contents present two semicircles at the high and low frequency regions measured at V = V_OC_ under dark conditions (Figure 8). We used an equivalent-circuit model, as inserted in Figure 8, to explain the EIS responses. R1 is the external series resistance including contacts resistance, CPE1 is related to the capacitive element at high-frequency response related to accumulation of surface charges at the interface of perovskite layer, R2 represents the charge transport resistance of the bulk perovskite film but contains information about the transport resistance of POSS passivation layer as well, and CPE2 represents the low-frequency capacitive related to the charge and defects accumulation in the film but can also be influenced from the ion in-diffusion recombination at the bulk of the film. R3 is the resistance related to the charge recombination at the bulk of the film due to presence of bulk defects and traps [30,31,32,33]. The fitting results are summarized in Table 1. With the increase in POSS content (POSS-0.005, POSS-0.01), the R2 and R3 increase, indicating that the charge recombination resistance increases but the charge transport resistance also increases. According to SEM morphology and absorption spectra analyses (Figure 2 and Figure 4), the decrease in charge recombination may be attributed to the increase in the grain size of MAPbI_3_. Larger grains and lower grain boundaries and thus reduce charge recombination at interfaces, consequently obtaining a higher J_SC_ [34,35]. We infer that POSS passivation enhances the MAPbI_3_ crystal growth and thus blocks the ion-diffusion and defect/trap-related recombination in the perovskite layer, but also increases the resistance at the interface between the perovskite layer and the HTL. The increase in charge recombination resistance by POSS passivation is also observed by dark current analysis (Figure S4). The POSS-passivated cell displays a smaller leak current and dark current than the control cell, implying that the POSS passivation suppresses the charge recombination and induces a strong depleted electric field. Too many POSS contents may lead to the increase in pinhole, which increases the charge recombination rate but decreases the charge transport resistance. Figure 9 shows the IPCE spectra and integrated J_SC_ of the control cell and the PSCs with various POSS contents. The values of the integrated J_SC_ agreed well with those obtained from J–V measurement. The curves present a cutoff at ~790 nm, corresponding to a bandgap of 1.57 eV for a typical MAPbI_3_ solar cell [36]. POSS passivation influences mainly external quantum efficiency in the wavelength range of 380 to 760 nm. IPCE spectra of the PSCs support the J–V observation and the aforementioned argument. The POSS-0.01 displays the highest quantum efficiency and the highest J_SC_.

## 4. Conclusions

In summary, this is the first attempt to use POSS as a passivation layer between the hole transport layer (NiO_X_) and the perovskite layer (MAPbI_3_) of PSCs. POSS passivation can make the surface of the NiO_X_ layer more hydrophobic and smoother surface, which leads to a significant increase in the MAPbI_3_ grain size and a slight increase in V_OC_. However, in addition to the increase in interface resistance, too much POSS makes the surface of NiO_X_ become too hydrophobic to spread over the perovskite solution and thus results in pinholes and a small grain size. A 0.01-mg·mL^−1^ POSS passivation displays the best performance, which increases the J_SC_ from 18.0 to 20.5 mA·cm^−2^ and an enhancement of 17.3% for PCE. The performance improvement arises mainly from the decrease in the rate of charge recombination.

## Figures and Tables

**Table 1:** The photovoltaic characteristics of the control cell and the PSC fabricated with various POSS contents.

Samples	POSS Concentration mg·mL^−1^	Voc, V	Jsc, mA·cm^−2^	FF, %	PCE (Average PCE), %	R2, Ω	R3, Ω
Control	0	1.05	18.0	70.18	13.3 (12.55 ± 0.49)	163.9	328.9
POSS-0.005	0.005	1.05	18.4	73.78	14.1 (12.64 ± 1.23)	209.2	506.4
POSS-0.01	0.01	1.07	20.5	71.33	15.6 (14.75 ± 0.71)	234.3	869.9
POSS-0.015	0.015	1.07	19.2	66.16	13.7 (12.85 ± 0.35)	193.7	440.3
POSS-0.05	0.05	1.06	15.2	71.60	11.5 (10.91 ± 0.62)	179.3	332.7

## Data Availability

The data presented in this study are available on request from the corresponding author.

## Supplementary Materials

The following are available online at https://www.mdpi.com/article/10.3390/nano11040974/s1, Figure S1: Schematic representation of PSCs with POSS passivation; Figure S2: Average grain sizes of the MAPbI3 layers over various FTO/NiOX/POSS substrates, Figure S3: AFM topography images of the FTO/NiOX/POSS with various POSS contents; Figure S4: Dark current-voltage curves of the control cell and the champion cell.
