# Supplementary material for: Fabrication and Characterization of an Efficient Inverted Perovskite Solar Cells with POSS Passivating Hole Transport Layer
Source: Nanomaterials (Basel). 2021 Apr 10;11(4):974. doi: 10.3390/nano11040974 (PMC8068981; doi:10.3390/nano11040974)
Supplement: Supplementary file 1 [file nanomaterials-11-00974-s001.pdf]

Supporting Information

Fabrication and Characterization of an Efficient Inverted Perovskite Solar Cells with POSS Passivating Hole Transport Layer

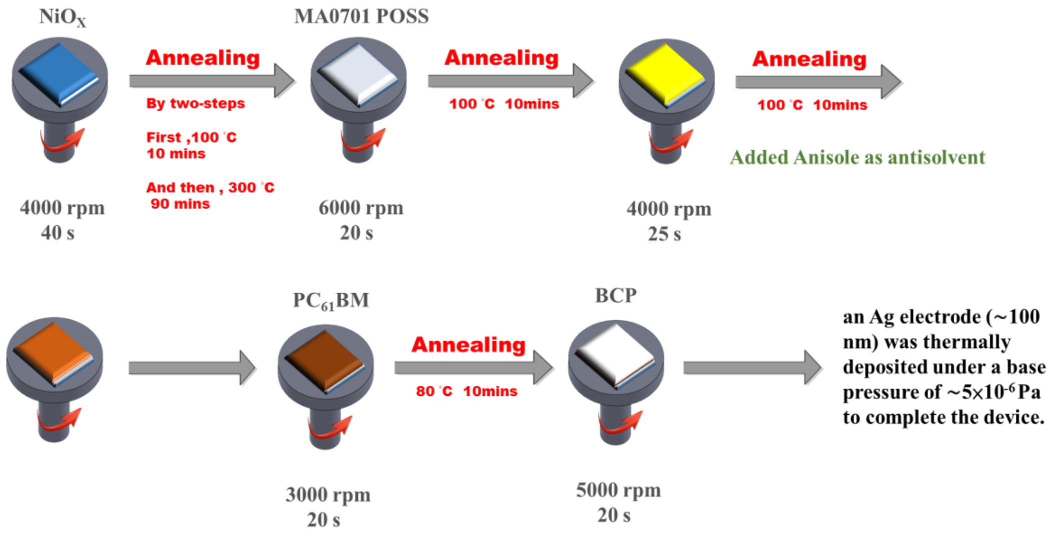

Figure S1. Schematic representation of PSCs with POSS passivation.

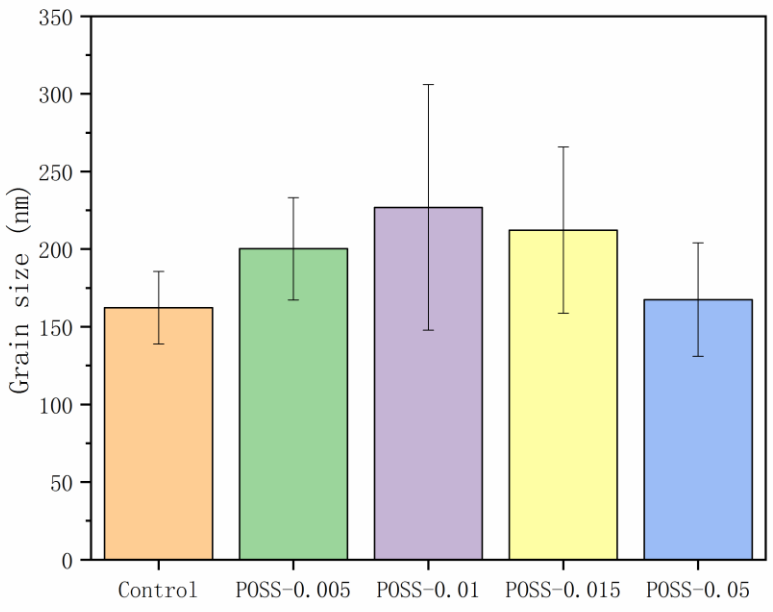

Figure S2. Average grain sizes of the $MAPbI_3$ layers over various FTO/NiO_X/POSS substrates.

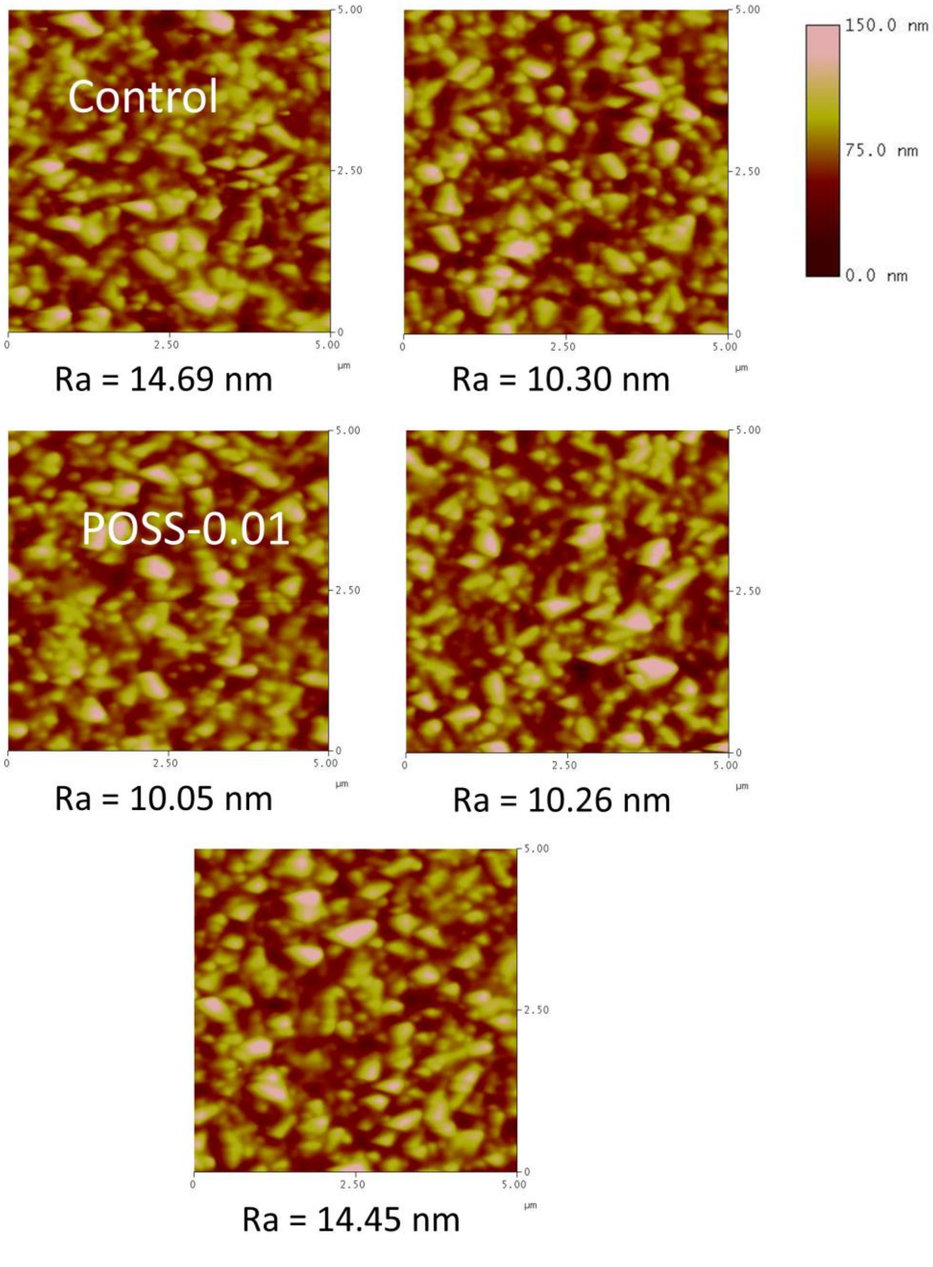

Figure S3. AFM topography images of the FTO/NiO_X/POSS with various POSS contents.

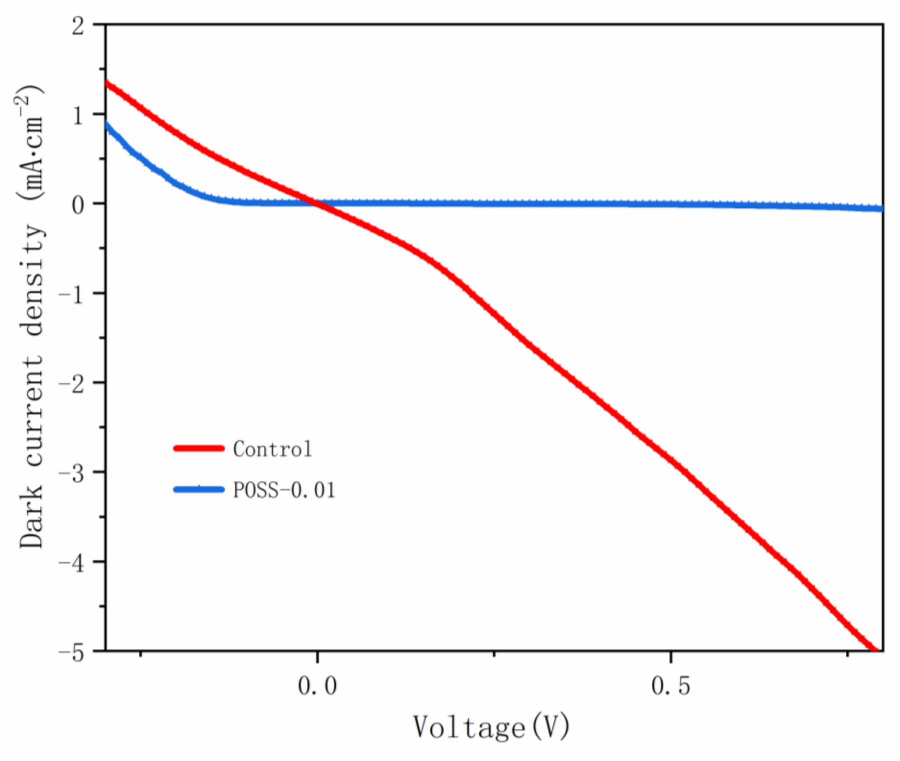

Figure S4. Dark current-voltage curves of the control cell and the champion cell.
